# Decision-Making in Agent-Based Models of Migration: State of the Art and Challenges

**DOI:** 10.1007/s10680-015-9362-0

**Published:** 2016-02-15

**Authors:** Anna Klabunde, Frans Willekens

**Affiliations:** Max Planck Institute for Demographic Research, Konrad-Zuse-Str. 1, 18057 Rostock, Germany

**Keywords:** Agent-based model, ABM, Migration, Decision-making, Choice theory, Microsimulation, Review

## Abstract

We review agent-based models (ABM) of human migration with respect to their decision-making rules. The most prominent behavioural theories used as decision rules are the random utility theory, as implemented in the discrete choice model, and the theory of planned behaviour. We identify the critical choices that must be made in developing an ABM, namely the modelling of decision processes and social networks. We also discuss two challenges that hamper the widespread use of ABM in the study of migration and, more broadly, demography and the social sciences: (a) the choice and the operationalisation of a behavioural theory (decision-making and social interaction) and (b) the selection of empirical evidence to validate the model. We offer advice on how these challenges might be overcome.

## Introduction

Migration modelling has a long history. For decades, the literature was dominated by the gravity model, which posits that the volume of migration between two locations increases with population sizes in each location and decreases with geographical distance between the locations. The spatial interaction models that evolved from the gravity model incorporated additional determinants of migration and historical migration patterns that were considered to have long-term effects (Sen and Smith 1995). The aim of migration modelling has been and still is to explain observed migration flows and to predict migration flows at a future point in time.

Although the level of migration between two locations may be related to push factors in the location of origin, pull factors in the location of destination, and intervening factors between the two locations (Lee 1966), the level of migration is ultimately the outcome of individual actions. Spatial interaction models do not capture individual actions; instead, they summarise the outcome of these actions. Microsimulation models take the individual as the unit of analysis and allow researchers to differentiate between individual characteristics and idiosyncrasies. These models provide a point of departure for modelling individual actions as outcomes of decision processes (see, e.g. Klevmarken 1997; Billari and Prskawetz 2003; Murphy 2003; Klevmarken and Lindgren 2008; Birkin and Wu 2012; Tanton and Edwards 2013). Although most microsimulation models refer to individual decisions, they are not “very explicit and detailed about the path economic subjects follow to reach a decision” (Klevmarken 2008, p. 33).

Decision-making is emphasised in behavioural models of migration. In these models, an individual is likely to leave a location if he/she expects to be better off elsewhere, and the barriers to migration are manageable. The attractiveness of a location is measured in terms of utility (e.g. in a utility maximisation model) or value (e.g. in a value expectancy model). Behavioural models were initially developed for an average individual and were later extended to account for observed and unobserved differences between individuals. In most of the behavioural models used today, the outcome of migration is uncertain. Utility and value are random variables with probability distributions. In most of the models, migration is triggered by the expected utility or the expected value. In some of the models, migration is embedded in the life course of the individual. Life events—such as marriage, divorce, graduation, or retirement—can change the utility of locations and may therefore trigger migration (Courgeau 1985). As migration has an impact on other life events as well, there is growing interest in modelling migration and the individual life course simultaneously. Furthermore, behavioural models often consider other individuals and institutions and their effects on migration. These other individuals—who may be household members, family members, friends, or members of the same community—may facilitate or inhibit migration and may influence both the timing of migration and the choice of destination.

In recent decades, migration models have become increasingly realistic by incorporating decision processes. That progress continues with the introduction of agent-based models (ABMs). In agent-based modelling, the focus is on individual agents, their decision processes, their interaction with other agents, and the effects of that interaction on decision processes. Differences between individuals can be introduced easily because agent-based models are microsimulation models at their core. Whereas in microsimulation models transition rates vary between heterogeneous individuals, in agent-based models decision rules can vary as well, depending, for example, on the reason for migration. Individuals who strive for a higher standard of living may be assumed to make decisions differently from those fleeing from war or political persecution.

Interactions between individuals often produce nonlinear effects at the population level. Agent-based modelling is the only method that allows for the explicit modelling of social interaction and the social networks that result from it. This represents a huge opportunity for modelling migration, as networks shape the migration decision, and especially the destination choice. Information on migration options and job market opportunities is transmitted through these networks. Moreover, networks represent a source of social capital, which can manifest itself in the form of financial aid to help cover the costs of migration or financial shortfalls immediately after migration. Network ties to the host country can thus facilitate the job search, provide initial housing, and serve as insurance to mitigate the enormous risk associated with migration. Network ties change through integration and assimilation, and they tend to become weaker over time and distance. Thus, these ties also influence how long migrants stay and whether and when they return to their home country. Networks are important for explaining population-level phenomena, such as the distribution of migrants within a country. Migration flows are often path-dependent and self-reinforcing processes that originate with interactions on the microlevel. Agent-based modelling can capture these processes explicitly and allow for the exploration of different mechanisms of social influence.

In demography, agent-based modelling has been applied most frequently in studies of partnership and marriage markets (Billari et al. 2007; Todd et al. 2005; Hills and Todd 2008; Bijak et al. 2013). Noble et al. (2012) studied the implications of household formation and of other demographic processes for the social care situation in the UK using an agent-based model. Aparicio Diaz (2010) and Aparicio Diaz et al. (2011) investigated the effect of the social network structure on marriage and transition to parenthood. Fent et al. (2013) used an agent-based model to study the impact of social structure and policies on fertility.

In this paper, we review agent-based models of migration, with a focus on the behavioural theories chosen. Migrations represent a subset of relocations, namely relocations that involve a change of residence beyond an administrative boundary. We exclude models of residential mobility or of mobility initiated by a desire for better housing. We do, however, include models of return migration (Klabunde 2014; Massey and Zenteno 1999; Biondo et al. 2013). Our review of the models shows that agent-based modelling is still in its infancy with regard to migration. There are a large number of models, but these models differ considerably in scale, scope, and documentation, in part because they originated in different disciplines, ranging from computer science to psychology. Moreover, most of these models were constructed from scratch, with little or no accumulation of knowledge. However, the review also found some striking commonalities in the design principles used by the developers of these models.

The structure of the paper is as follows. In Sect. 2, we briefly introduce agent-based modelling and define it for our purposes. In Sect. 3, we summarise the state of the art in agent-based models of migration, with a special emphasis on the decision-making rules chosen. In Sect. 4, we list and discuss several of the choices that are typically made in specifying a model. In Sect. 5, we point out two challenges that may arise in agent-based modelling. Finally, in Sect. 6, we discuss our results and suggest a future course for researchers interested in developing agent-based models of migration.

## Definition and Search Method

Despite some claims to the contrary, defining an agent-based model is not an easy task. There is a continuum of model types, and decisions about whether a model is a microsimulation or an agent-based model are sometimes arbitrary. For a discussion of the differences between agent-based models and microsimulation models, see, e.g. Siebers et al. (2010), Squazzoni et al. (2013), and Richiardi (2014).

Classical analytical or statistical models focus on the aggregate or the population level in demography. Microsimulation focuses on individuals and their actions, and the characteristics of the aggregate are obtained by pooling individuals. Actions usually imply transitions in the life course. Rates (or probabilities) of transition vary between individuals with different attributes and experiences. Individuals with equal transition rates may behave differently because of chance and chance only. The influence of chance is operationalised by drawing a random number from a probability distribution.

Agent-based models focus on individuals, as well. ABMs are very similar to microsimulation models, but they differ in one important respect: the transitions are determined not by transition rates, but by causal mechanisms. The mechanisms refer to how individuals decide how to act and when to act. Individuals follow different procedures (rules) in choosing among alternatives. Most ABMs consider explicitly the influences of the people with whom the individual interacts. A critical part of agent-based modelling is the description of the mechanisms of decision-making and the mechanisms of social interaction. It is in this stage of the modelling process that theories of choice, action, and social interaction are needed. The extent to which behavioural theories are used in ABMs varies greatly, as we will show in this paper. Random factors still cause decision processes and social interaction processes to vary between identical individuals, even if they are in the same context.

Because microsimulation and ABMs are closely related, several authors have called for a removal of the barriers that continue to exist between the two fields (see, e.g. Richiardi 2014; Siebers et al. 2010). We follow this suggestion and avoid making clear distinctions between the two, as these divisions strike us as being somewhat artificial. Thus, some of the models we consider ABMs and include in this review might be called microsimulations by others.

Our definition of agent-based model is centred around the concept of “agent” (Macal and North 2010). Agents are discrete entities that are autonomous and capable of *making decisions* using procedures or *rules.* Autonomous means that they can act *independently*. Other agents may influence their decisions, however. Agents have *goals*, at least implicitly. The aim of an agent-based model is to uncover *causal mechanisms*. ABMs and microsimulations both lack equations which govern the overall social structure on the macrolevel (Axtell and Epstein 2006). Structures at the macrolevel emerge from actions and interactions at the microlevel. This fairly broad definition of the ABM is debatable, but is in line with many definitions in the literature (Epstein 2006; Tesfatsion 2002; Gilbert and Troitzsch 2005).

We followed a systematic search procedure in two steps to ensure we did not miss any relevant work. As a first step, a search was performed on Web of Science using the following combination of keywords: Topic = (agent-based model*OR agent-based simulation) AND Topic = (migration) NOT Topic = (cancer OR medicine OR tumour OR disease OR therap*) NOT Topic = (server OR sensor) NOT Topic = (chemi*OR biolog*OR mineral*OR seismic*). The latter criteria of exclusion had to be used because a large number of the publications were about the migration of cancer cells or minerals or were from the field of engineering. This approach yielded 291 results, of which 22 turned out to be relevant to us following the above definition. Second, we added papers from our personal archives with which we were familiar and which were not found during the search procedure. These papers were not found either because the authors did not call their methodology “agent-based” (e.g. Massey and Zenteno 1999), even though it fit our definition, or because the publication was in the form of a working paper, dissertation, or book and was thus not covered by Web of Science. The second step yielded seven papers. We excluded papers in which the description of the migration decision was not clear enough to allow for categorisation or analysis (e.g. Wu et al. 2011a, b; Spencer 2012; Ruiz et al. 2010, 2014). We also excluded “random mobility” models, in which migration was considered completely random and without purpose (see, e.g. Chiong and Kirley 2013). An overview of other aspects that were covered in the papers we reviewed can be found in Table 2 in the Appendix.

## Theories of Decision-Making in ABMs of Migration

In this section, six types of models are distinguished. The first type makes no or minimal use of decision theory. The main purpose of the models in this category is to show that the interaction between individuals using simple behavioural rules can generate complex patterns at the population level. The second type of model uses microeconomic expected utility theory to explain the choices people make between discrete alternatives. The third type of model is based on a theory of action derived from social psychology. The fourth type uses heuristics. The fifth type is loosely based on decision theory and relies more heavily on direct observation. The last type relies exclusively on direct observations.

We evaluate these decision theories in terms of their adequacy for modelling decision-making in agent-based models of migration. In our evaluation, we use eight criteria that we believe reflect both the various aspects of empirical migration decision-making and the requirements for computational modelling. These criteria are:

1. The theory should allow for the possibility that there is a gap between desires or intentions and actual behaviour.
2. The theory should take into account social influence. It has often been shown that migrants are influenced by the choices of others and that they depend on others for help (Haug 2008; Munshi 2003).
3. The theory should allow for uncertainty. The decision to migrate is made under conditions of uncertainty.
4. The theory should be able to situate the migration decision in the life course and to relate it to other demographic events and changes in goals.
5. The theory should allow for the time it takes to plan a migration. In most cases, migrants spend long periods planning and preparing for migration, and during these periods, demographic events might occur and the labour market situation in the home or the host country might change. Ideally, it should be possible to allow for this temporal dimension of the decision and the effects of intervening events.
6. The decision rules laid out in the theory should be based on decision theory and empirical evidence.
7. The theory should be as simple as possible and as complex as necessary.
8. The theory should be falsifiable in principle. If the model outcomes at an aggregated level are not in line with empirical observations, the assumed decision behaviour is unlikely to describe the actual data-generating process.

We evaluate the decision theories as they have been used in agent-based models of migration. In some cases, it is possible to fulfil additional criteria by extending the decision behaviour or by focusing on different aspects of the decision. Table 3 in the Appendix provides an overview of our evaluation based on these criteria.

### Minimalist Models

The purest example of a minimalist model is that of Schweitzer (1998), who studied migration and the resulting economic agglomeration in space. The agents in his model are active Brownian particles that have two different internal states (employed and unemployed). Employed agents generate a field around themselves, the force of which depends on the wage. This field attracts unemployed agents who move towards it.

Schweitzer compared his results to those of Krugman (1992), who studied the same problem. Schweitzer was not interested in any particular behavioural theory, but rather in outcomes on the macrolevel and the basic rules that generate these outcomes. Jiang et al. (2010) developed a variant of Schweitzer’s model by adding the influence of the cultural difference. Although the model is essentially the same as Schweitzer’s, the agents’ actions are motivated by utility maximisation.

Silveira et al. (2006) is a limiting case. While the authors call their approach a statistical mechanics Ising model, their agents are utility maximisers. The model is essentially a Harris–Todaro type model of rural–urban labour migration. The authors showed the parameter settings under which the equilibrium condition from Harris and Todaro (1970)—namely, the equalisation of expected wages in the rural and the urban sectors—can be brought about. The model of El Saadi et al. (2010) is very similar in that it is essentially a discretisation of the differential equation model by Todaro (1969). The aim of minimalist models, as in these four cases, is usually to show how macrooutcomes can be “grown” from very simple microlevel rules and thus to show which minimal assumptions are necessary to generate the observed outcomes.

Ichinose et al. (2013) used game theory to study the impact of migration on cooperation. Their aim was not to explain migration, but rather to evaluate the effects of migration on the evolution of cooperation. They posited that migration is governed by a simple rule: i.e. initially, individuals are randomly distributed over space and categorised as co-operators or defectors, and an individual moves when the share of defectors among his/her neighbours is high. After migration, the individual engages in a prisoner’s dilemma game with his/her new neighbours. The purpose of that game with the neighbours in the destination area is to learn the best strategy (i.e. to cooperate or to defect). When the game is over, the individual imitates the strategy of the neighbour with the highest pay-off. The authors found that long-range migration enhances cooperation.

The great advantage of minimalist behavioural models is their simplicity, but the empirical relevance of the decision rules employed in these models is questionable.

### Microeconomic Expected Utility Maximisation

The model by Heiland (2003) is the agent-based model of migration that is most closely aligned with standard economic theory. Because the agents in the model do not interact, it could be argued that it is not an ABM at all. Yet, we decided to include it because it fulfils the other criteria and is thus an interesting borderline case. Heiland used the model to study the migration of East Germans to West Germany after German unification.

The potential migrants in the model perform finite horizon and discrete time expected utility maximisation with rational expectations. The control variable is the location choice, and the state variables are employment status and location. Utility is derived from the consumption opportunities and the amenities at the location, the evaluation of which will differ between individuals (apart from the initial location, this is the only point of heterogeneity). The expectation is formed in the first period, during which individuals correctly predict—given some stochastic influence—their employment probabilities, income, search costs, and migration costs. The optimal decision rules are derived by backward induction using a dynamic programming algorithm. Heiland (2003) demonstrated that geographic proximity and the differences in the demand for technically skilled labour across western German states largely explain the distribution of migrants across states. His model also accurately predicted the decrease in migration over the years.

The model by Biondo et al. (2013) is the only model presented here that is only about return migration. In the model, the agents maximise income, for which social capital serves as a proxy. Social capital increases with the duration of stay. Espíndola et al. (2006) developed a model of pure income maximisation: as in Silveira et al. (2006), the agents choose the state (rural or urban) which they expect will yield the highest income. *Information acquisition* occurs through a comparison with the neighbours’ earnings: the agent changes his/her state if his/her income is lower than the average of the neighbours’ incomes. The equilibrium is reached when the expected wages are equal in rural and urban areas, as is postulated in the model by Harris and Todaro (1970). The set-ups and topics of the models by García-Díaz and Moreno-Monroy (2012) are somewhat similar: the agents occupy states representing the rural, informal urban, and formal urban sectors. However, the agents have perfect information about all of the macrolevel variables. Their rationality is not bounded in any way. Workers leave their current state with a fixed probability; the only actual decision is which sector they will move to. This is done via utility maximisation: utility is an additive function of the expected wages and the number of neighbours at the same “location”. The probability of choosing a location is then formulated as a discrete choice model (logit model). The main result of García-Díaz and Moreno-Monroy (2012) is that an increase in social influence delays convergence to a steady state.

### Psycho-Social and Cognitive Models

The title of this section is borrowed from An (2012). It is something of a catch-all, as it covers a wide range of theories from (social) psychology. Three migration ABMs fall into this category. The first is the model by Kniveton et al. (2011, 2012) based on the theory of planned behaviour. One of the authors of the first model developed another model that is also based on the theory of planned behaviour (Smith 2014). The third model, by Reichlová (2005), is based on Maslow’s motivation theory.

The theory of planned behaviour (Ajzen 1991, 2004) is an extension of the theory of reasoned action by Fishbein and Ajzen (1975) and Ajzen and Fishbein (1980). Individuals form attitudes towards a certain behaviour (in this case, migration), which are defined as evaluations of different outcomes of the action, weighted by their subjective probability of occurrence. Individuals have subjective norms that can differ in relevance depending on the source. Moreover, individuals attach a certain level of perceived behavioural control (PBC) to an action, that is, they calculate a subjective probability of actually being able to perform an action. Attitudes, subjective norms, and PBC jointly determine the individual’s intention. Whether the behaviour occurs depends on the individual’s actual control over it. For a recent presentation of the theory, see Fishbein and Ajzen (2010) and Ajzen and Klobas (2013).

Kniveton et al. (2011) studied climate-driven migration in Burkina Faso. According to their model, individuals develop attitudes towards migration which depend on the probability of migration of similar individuals (based on age, gender, and marital status). These probabilities are estimated from a household survey. The transition from developing an attitude to formulating an intention to migrate is an outcome of a decision process. Other individuals influence the decision to migrate (“subjective norms”). The subjective norm is a function of “positive messages”. For details, see Kniveton et al. (2012).^1^ PBC is computed as the sum of a person’s assets and migration experience, scaled to be between zero and one. If a random number drawn from a uniform distribution is less than the PBC value, then the PBC is set to one; otherwise, it is set to zero. If the PBC is zero, the individual gives up his/her initial intention to migrate. Thus, if an individual does not consider migration to be within his/her behavioural control, he/she will not develop an intention. All of the behavioural options (i.e. to stay home or to migrate to one of the possible destinations) receive an intention value; the one with the highest value is chosen. An interesting emergent result of Kniveton et al. (2012) is that population growth enhances the impact of climate change on migration. This is because the network effects increase as populations grow. Smith (2014) employed essentially the same decision model in his study on rainfall-induced migration in Tanzania.

Reichlová (2005) developed a migration model that is based on Maslow’s (1954) theory of the hierarchy of needs. Migration is influenced stepwise by income, safety, and social needs. The study tried to explain why there is surprisingly little migration within Europe, despite persistent income differences, and why wages do not therefore equalise.

Psycho-social models, and particularly the theory of planned behaviour, allow for the inclusion of a large number of the features considered relevant for the migration decision, especially the distinction between desired and actual behaviour, but also social influence, the role of uncertainty, and the treatment of migration together with other life events. The empirical relevance of the theory of planned behaviour for a behaviour with similar far-reaching and uncertain consequences—i.e. motherhood—has been shown by Ajzen and Klobas (2013). However, psycho-social decision theories tend to be complex and can be criticised for being arbitrary as they theoretically allow for the inclusion of an infinite number of decisive factors and beliefs.

### Heuristics Without Direct Empirical Correspondence

According to Gigerenzer and Gaissmaier (2011), a heuristic is “a strategy that ignores part of the information, with the goal of making decisions more quickly, frugally, and/or accurately than more complex methods” (p. 454). In many situations in which they make decisions, people rely on heuristics, rather than on rational decision-making processes based on full information (Tversky and Kahneman 1974; Shah and Oppenheimer 2008; Newell et al. 2003).

In the model of historic human settlement in arid environments (Janssen 2010), an agent decides to migrate when he does not receive the minimum level of required resources. Janssen found that climate variability increases system resilience. Rogers et al. (2011) showed that people living in stratified, unequal societies have a greater tendency to migrate and thus to conquer or displace people living in egalitarian societies over time. Migration is triggered by simple thresholds: people migrate when the population in their community declines sharply, the resources in their area fall below a certain level, or their income falls below a certain level. Highly stylised models without direct empirical correspondence often use heuristics, too. Hafizoglu and Sen (2012), who studied the spread and distribution of opinions when agents can adapt or migrate, is an example. Agents decide whether to stay or to migrate based on the gap between their own opinion and the views of the majority in their current community.

Like minimalist models, heuristics tend to be simple and easily falsifiable and to allow for social influence. Accounting for uncertainty or other demographic events is easier in heuristics than in minimalist models. Nevertheless, heuristics are limited, since, by definition, they stop being heuristics once the decision rule becomes more complex. In reality, the migration decision is almost certain to be complex.

### Based on Decision Theory and Direct Observation

The models by Rehm (2012) and Klabunde (2014) use utility maximisation. In Rehm (2012), who studied migration from rural Ecuador to urban Ecuador and to New York, the behavioural motives are drawn mostly from the economic migration literature, and utility maximisation is implicit. In the baseline version of her model, migration propensities and destination choice are influenced by the location of an individual’s family members, the total number of migrants at a destination, whether the individual receives remittances, the individual’s assets, the availability of jobs at the destination, and the individual’s income, implemented through multinomial logit. Her aim was to distinguish between different theories of remittances by determining which behavioural rules produce certain stylised facts on the macrolevel, such as the distribution of people across the three locations and the distribution of wealth across individuals. This is an extremely interesting and promising research strategy and one that is unique to agent-based models. She found that no behavioural rule in isolation is able to reproduce all of the stylised facts at the same time.

Klabunde (2014) studied circular migration. Her model is halfway between the economic ABMs described above and the empirical models described in the next section. Her choice of behavioural motives was also based on the economic literature, and she calibrated the coefficients in the behavioural rules using a large microsurvey data set, the Mexican Migration Project. The decision to migrate is determined by the individual’s expected earnings, wealth, age, and network ties to other migrants, whereas the return decision is influenced by the number and the strength of ties to the home country, as well as age. Klabunde (2014) found that the distribution of migrants across US cities in the data follows a power-law distribution, whereas the distribution of the number of trips across migrants is negative-binomial.

The study by Massey and Zenteno (1999) on Mexican–US migration is close to being a pure microsimulation study. It contains some interactions and behavioural motivations from utility theory, which is why it is included here. The probability of migration and of return migration in a person-year were considered functions of an individual’s age, sex, number of previous trips, and number of years spent abroad, as well as of the number of trips made by other community members and the number of years they spent abroad. The parameters of the heuristic were estimated from the Mexican Migration Project data set via logit regression. They found that not taking account of social network effects results in a considerable underestimation of the size of Mexican population living in the USA.

Naqvi and Rehm (2014) modelled the response of low-income agents to natural disasters; one of these responses is migration. They found that increased migration to the cities in response to a drought leads to a decline in urban incomes, which in turn causes the demand for food to rise, and already high food prices to increase further.

The aim of combining decision theories with other empirical rules is to combine the rigour of a decision theory with the empirical accuracy of observational rules. While this works to some extent, it comes at a cost, as the decision rules are no longer easily falsifiable. The fact that stylised facts can be reproduced with a mixture of a theory and empirical rules does not increase the empirical weight of the theory. These types of models can be interesting case studies, but their generalisability is limited.

### Purely Empirical, Observational Rules Without Mention of a Theory

There are several empirical models that do not mention any particular theory. The choices of behavioural rules seem to be entirely empirically motivated: the determinants of migration are estimated from data through statistical and econometric analysis, or they are taken from expert or stakeholder interviews.

Cai and Oppenheimer (2013) provided an example of such a model. They studied climate-induced migration and distinguished between intentions and behaviour. According to this model, the individual’s intention to migrate is influenced by crop yield, gender, age, assets, migration experience, risk attitude, and social network, linked together in a logistic regression. Intentions are converted to behaviour by drawing a random value from a standard uniform distribution. If the random number is smaller than the probability of developing an intention to migrate, the individual migrates; otherwise, the individual stays. This method implies that the proportion of people migrating is the same as the proportion developing an intention to migrate. This approach is fairly common; e.g. Mena et al. (2011) employ it as well.

Migration is just one of many behavioural options in the model by Berman et al. (2004), which seeks to explain how people in one particular village in the Yukon, Canada, adjust over the long run to climate change and new economic opportunities. The possible actions are hunting, looking for a job, or migration. This model is a typical example of a case-based model in the taxonomy by Boero and Squazzoni (2005). The choice between the alternatives is not motivated by any specific theory, but rather by different empirical studies and qualitative interviews with community members and experts.

Naivinit et al. (2010) applied companion modelling to determine the decision rules related to seasonal and permanent labour migration among rice farmers in north-east Thailand. The purpose of their study was to evaluate the likely impact of different irrigation policies. The decision process is a sequence of if–else-type questions resulting in one of the three possible options: seasonal migration, permanent migration, and no migration. Smajgl and Bohensky (2013) followed a very similar strategy.

The big advantage of empirical rules is, of course, their empirical accuracy. One of the disadvantages is a lack of guidance on which factors to include; potentially, anything could be included which is statistically significant. But this makes for overly complex decision rules with little meaning. Of course the problem is that the more variables that are included, the better a behaviour can be explained in one particular situation, but the less generalisable the explanation is, and the less likely it is to be of value in a different situation.

## Modelling Decision-Making

The number of options for modelling decision-making is almost infinite. The modeller has to make a choice in several dimensions, four of which are addressed in this section. The first is how expectations are formed and how information is gathered to form expectations. The second is how alternatives are evaluated and how one alternative is selected. The third is how to determine the complexity of the model describing the decision process. The final choice is at what level of detail a migration network should be modelled.

### How are Expectations Formed?

This question can be split up into two separate ones: How much and what kind of information do agents have about the present, and how do they use information to predict the future? On one end of the spectrum, agents might have perfect information about the present—i.e. they know all of the other agents’ variables and their own state variables, everyone’s expectations, and the true state of their environment. If the environment is simple enough—e.g. if the time series are stationary, if the stochastic disturbance terms are well behaved, and, most importantly, others behave just like they do—it is possible to predict the future net of stochastic disturbances, and agents can form *rational expectations* and base their behaviour on those expectations. If everyone were to use this decision rule, the future state of the world would be exactly the state of the world everyone has based their expectations on. This set of assumptions was implemented in Heiland (2003). The agents in García-Díaz and Moreno-Monroy (2012) have perfect information as well, but their decisions are not intertemporal.

The other end of the spectrum would be an agent with no information at all, or with very little, purely local information, like in Schweitzer (1998). With (almost) no information, there can only be period-by-period decision-making in which no expectation of the future is formed at all. Most ABMs lie somewhere in between. Agents usually have some information about the current state of the world and use some way of extrapolating into the future in order to form an expectation about how their decision will affect their future well-being. If agents have information on the correct value of a relevant variable in the present, the easiest way to extrapolate is to assume that the relevant variables are going to remain constant in the future and to base their decision on this assumption (Reichlová 2005; Kniveton et al. 2011, 2012; Naqvi and Rehm 2014). If the information is not perfect—e.g. if it is only local—one way for an individual to form an expectation is to observe the state variables (e.g. location) and the corresponding level of satisfaction (e.g. happiness or income) of the agents the individual can access and to assume that if he/she chooses the same state, he/she will achieve the same or at least a similar level of happiness (Espíndola et al. 2006; Barbosa Filho et al. 2011; Klabunde 2014). A different way of forming expectations is for the individual to compare himself/herself not to others, but to his/her own past experience (Silveira et al. 2006).

### How are Choices Evaluated?

Once agents have assembled a complete set of choices, one of the options has to be chosen based on an evaluation of these choices. In the migration context, the different options are usually migrating or not, or migration to different destinations. The easiest way to evaluate choices is to assign numbers to options and then to choose the option with the highest valuation (Kniveton et al. 2011, 2012; Reichlová 2005). Alternatively, an action may be triggered deterministically once a functional output exceeds some threshold value (Hassani-Mahmooei and Parris 2012; Biondo et al. 2013; Barbosa Filho et al. 2011; Espíndola et al. 2006). Very often, however, some stochastic element in decision-making is assumed; thus, many authors employ a binomial (Massey and Zenteno 1999; Silveira et al. 2006) or multinomial (Rehm 2012; García-Díaz and Moreno-Monroy 2012; Heiland 2003; Hafizoglu and Sen 2012) logit implementation.

### How Complex is the Decision? Does it Involve Several Steps?

The complexity of decision-making in reality depends on the scope of the possible outcomes of an action. Generally, the greater the impact the decision is expected to have on personal satisfaction, the more cognitive effort is involved in making the decision (Janssen and Jager 2001). Thus, the migration decision is usually very complex in reality, and all of the model representations are necessarily simplifications. Additionally, in reality as well as in models, the sophistication of decision-making depends on the availability of information and thus on the capacity of the agent to reduce uncertainty.

The complexity of decision-making in all models, and not only in ABMs, seems to be an inverted u-shaped function of the information available to the agents. Hence, the agents in Schweitzer (1998), who have only local information, mechanically move in the direction of the largest wage gradient; this is an extremely simple decision rule. Agents with some but not perfect information are fairly sophisticated in their decision-making; the application of the theory of planned behaviour in Kniveton et al. (2011, 2012) serves as an example. Agents with perfect information, like in Reichlová (2005) or Biondo et al. (2013), again tend to have very simple decision-making rules, such as utility maximisation. An exception is the combination of perfect information and perfect rationality in an *intertemporal* framework, such as that of Heiland (2003): rationality requires the agents to perform dynamic programming in their head, thereby computing the expected utilities associated with different decision paths over time and determining an optimal decision rule.

The migration decision often is composed of a “deliberate” cognitive action performed by the agent and a random component, which, for example, regulates whether a particular agent makes a decision at all in a given period (Silveira et al. 2006). The deliberate decision-making process often involves two steps: some kind of evaluation of the choices and the transformation of the results of this assessment into a decision or into a probability of taking action. This second step is often implemented in a logit framework. In the latter case, the third step of the decision-making is then a random draw to determine whether the agent performs the action.

Barbosa Filho et al. (2011) have their agents check several conditions before they reach a decision. The potential migrants in Klabunde (2014) first compute their wage expectations, then check whether the expected wage exceeds the current wage, then determine whether they can afford to migrate, and then decide whether to migrate based on a probabilistic rule. The potential migrants in Hassani-Mahmooei and Parris (2012) first compute the push factors of their home location, and then the intervening factors on the household level, such as property ownership and employment. If the sum of these criteria exceeds a threshold, the agent migrates.

### Are Networks Included? If so, What is Transmitted Through Them? Are They Exogenous or Endogenous to the Model?

The influence of other migrants and potential migrants on the migration decision has been found to be a determinant of migration and location choice in numerous studies (Haug 2008; Munshi 2003). Thus, many agent-based models include some kind of network or peer effects; indeed, these effects are often the reason why an ABM is chosen in the first place. Networks are the channel through which something is transmitted, e.g. information and/or social capital.

The simplest kind of network is a local interaction with the agent’s neighbours in a Moore neighbourhood, which consists of the eight cells surrounding a central cell in a two-dimensional square lattice. This framework is implemented in Espíndola et al. (2006), Silveira et al. (2006) and García-Díaz and Moreno-Monroy (2012), although in these studies the model grid does not represent geographical space, but rather a social space so that agents are spatially close to those individuals they are socially close to. In Espíndola et al. (2006), migrants gather information about wages from their neighbours, which might trigger migration out of pure income maximisation, whereas in Silveira et al. (2006) and García-Díaz and Moreno-Monroy (2012), social ties represent a kind of social capital that is only activated when two individuals are in the same state (e.g. rural or urban).

In Reichlová (2005), social capital is embodied in network ties, and agents are assumed to have a preference for being in the same region as their network neighbours. Network ties become stronger with each period in which the two agents are in each other’s Moore neighbourhood and become weaker otherwise. Thus, the network evolves endogenously with the migration behaviour. Klabunde (2014) modelled this process in much the same way, although in this case network ties serve as transmitters for information and, at the same time, as a representation of social capital. In Biondo et al. (2013), social capital represented by network ties is of utmost importance, as it serves as a proxy for income.

Massey and Zenteno (1999) is an example of an ABM in which the network ties are not explicit, but are represented by the collective migratory experience of other community members. The experience is an explanatory variable in a regression model, with the migration probability as the dependent variable. The functions and forms of networks in the ABMs discussed are summarised in Table 1.

**Table 1:** Functions and forms of social networks in agent-based models of migration

	Information transmission	Social capital
Fixed network	Espíndola et al. (2006), Kniveton et al. (2011, 2012) and Smith (2014)	Silveira et al. (2006) and García-Díaz and Moreno-Monroy (2012)
Evolving network	Klabunde (2014), Barbosa Filho et al. (2011), Rehm (2012) and Ichinose et al. (2013)	Klabunde (2014), Reichlová (2005), Barbosa Filho et al. (2011), Biondo et al. (2013), Rehm (2012) and Hafizoglu and Sen (2012)
Network only implicit		Massey and Zenteno (1999)
No network influence on migration	El Saadi et al. (2010), Naqvi and Rehm (2014), Janssen (2010), Schweitzer (1998), Jiang et al. (2010), Mena et al. (2011), Naivinit et al. (2010), Rogers et al. (2011) and Smajgl and Bohensky (2013)	

An elegant way to help ensure that the modeller is aware of all of the choices that have to be made is through the use of a protocol for the description of agent-based models, such as the ODD (overview, design concept, details), which was introduced by Grimm et al. (2006). Especially for models that involve human decision-making, the ODD + D protocol—in which the additional “D” stands for “decision”—seems to be a promising extension of the well-known ODD protocol (Müller et al. 2013). The modeller can use the prescribed format as a checklist to help her assess whether she has well-thought-out solutions for modelling every aspect of the migration decision discussed above.

## Challenges for Agent-Based Modellers

In this section, two challenges in the development of agent-based models in demography are singled out. The first challenge is the selection of a decision theory, and the second challenge is the determination of the role of data.

### Challenge 1: Which Decision Theory Should be Chosen?

In the review in Sect. 2, we saw that different decision theories have been used to model the migration decision. To some extent, the choice of a particular decision theory is driven by the authors’ background: while economists often use utility maximisation models, other social scientists are more likely to employ theories from cognitive psychology, and physicists tend to prefer minimalistic models. Several authors do not adhere to any decision theory at all, but instead use aspects from different theories in the decision rule. This often results in somewhat arbitrary behavioural rules. We suggest that researchers use one decision theory, such as utility maximisation or the theory of planned behaviour. In the theory of planned behaviour, for example, factors that were not originally part of the theory can be included: peer effects and the imitation of other migrants can be incorporated as social norms, and information received from others can be used to update the subjective probabilities of different outcomes that are needed for attitude computation.

Models that start with theory rather than with data have the advantage that they can go beyond mere extrapolation. By using a behavioural theory, we can hope to predict how agents will react to drastic changes in their conditions. When using pure time-series methods, that is not possible. There is another advantage of using theory: in situations in which no data or only inadequate data are available the implications of different theories can be tested in an agent-based model, and their respective predictions can be compared to real-world observable data (Silverman et al. 2011).

### Challenge 2: What is the Role of Empirical Data?

The ABMs reviewed above differ considerably in the amount of empirical data they use, ranging from none (e.g. Silveira et al. 2006; Espíndola et al. 2006) to a large amount (Klabunde 2014; Kniveton et al. 2011, 2012). A few existing studies have addressed the question of how much data are needed for various kinds of agent-based models (Boero and Squazzoni 2005; Brenner and Werker 2007; Janssen and Ostrom 2006).

Empirical data are used for estimation and validation. Estimation refers to the determination of the range of plausible values of parameters of the model and to the selection of the most acceptable parameter values. Those are the values that are the most likely or that minimise a function of the distance between observations and simulations (see, e.g. Kennedy and O’Hagan 2001; Grazzini and Richiardi 2015).

Validation refers to matching the model outcome and data. Cirillo and Gallegati (2012) have suggested a three-step procedure for model validation. They recommended first finding reasonable parameter ranges from the data, then qualitatively checking whether the model works as expected by comparing the model output with stylised facts, and, finally, estimating the parameters to find a good fit of the model.

Sensitivity analysis is an essential part of estimation and validation. Its purpose is to determine the parameters to which output is very sensitive. For some parameters, small changes may have large effects on the outcome of the model. In sensitivity analysis, it is generally not possible to run the model with every possible parameter combination. Factorial design is used to select combinations of parameters. Lorscheid et al. (2012) have provided guidelines. An important type of factorial design is the Latin hypercube; for an early description, see McKay et al. (1979); for more recent variants, see Pronzato and Müller (2012).

An alternative approach is to build a statistical meta-model or a model of the agent-based model that can be used to explore the parameter space much more efficiently. Gaussian emulators are examples of such meta-models. For a recent demographic application of a Gaussian emulator, see Bijak et al. (2013).

Table 2 in the Appendix provides information on the use of data in the models reviewed in this paper, i.e. whether and what kind of estimation or calibration procedure was used, whether a meta-model was used, and whether a sensitivity analysis was performed.

## Discussion

The aim of this paper has been twofold. First, we wanted to review the state of the art in agent-based modelling of migration with respect to the implementation of behaviour and to identify commonalities and differences in modelling decisions. Second, following a discussion of the main challenges in agent-based modelling in migration, we wanted to suggest possible directions for future work.

Regarding the first aim, we find that, apart from showing plausible data-generating mechanisms for stylised facts (Klabunde 2014; Heiland 2003; Rehm 2012), agent-based models of migration have mainly succeeded in finding ways to explicitly model network interaction, which has often been found to be an important determinant of migration decisions. Networks have been shown to amplify the effect of policies (Massey and Zenteno 1999) or climate change (Kniveton et al. 2012; Cai and Oppenheimer 2013) and to prolong the time to steady state, if there is one (Silveira et al. 2006; García-Díaz and Moreno-Monroy 2012). Moreover, ABMs allow researchers to make spatial predictions (Hassani-Mahmooei and Parris 2012) and to study distributions as both inputs and outcomes of interaction (Rehm 2012; Klabunde 2014).

We have reviewed different domains in which the modellers have had to decide how to model decision-making. We found that the ways in which expectations are formed in agent-based models of migration tend to be very simple: i.e. most agents just assume that today’s conditions will be valid in the future as well, or they expect that if they migrate they will receive the same wages as they observe their network neighbours receiving. Since having rational expectations in the economic sense is not an option if there is true interaction between heterogeneous individuals, as is the case in most migration models, this choice is reasonable. Nevertheless, more sophisticated ways of forming expectations should be considered in future research.

For the implementation of choice in ABMs, the formulation as a discrete choice problem in a logit and probit framework, which was pioneered by McFadden (1976, 1978), is the most common approach. The advantage of this approach is that it allows the modeller to transform the factors that influence behaviour into probabilities of action. The functional form produces output in the range [0,1], which makes it superior to a linear probability model, and because this approach can account for stochastic influence, it is superior to pure maximisation.

We categorise the ABMs of migration according to their use of networks: the transmission of both information and social capital through networks has been modelled. In some cases, the network is assumed to be fixed, and network ties do not change over the course of a simulation. In other cases, however, the network evolves through migration behaviour.

We now discuss the important issues that should be tackled in agent-based modelling of migration. First, the disciplinary barriers that currently exist should be removed. A good example is the recent extension of discrete choice theory, which is one of the main decision theories in economics and geography, with new applications in predictions of choice outcomes and effects of public policy. Recently, McFadden and other leading authors extended the discrete choice model “by including an explicit representation of the process and the context of decision-making” (Ben-Akiva et al. 2012). Process refers to the steps involved in decision-making. Context refers to factors that affect the process, in this case, social networks. Their framework includes most of the elements of the theory of planned behaviour, including subjective perceptions rather than objective measurements and the influences of other.

Second, the modelling of social networks in ABMs should take advantage of recent developments in social network research. An important challenge is to model precisely how social network effects emerge and affect decisions, and, more importantly, how they co-evolve. The full potential of studying the role of networks in migration that the methodology provides has not yet been exploited; it would, for example, be interesting to study the implications of different kinds of preferential or random attachment in networks or of different network topologies. Snijders et al. (2010) developed actor-based models of social network development that could be applied widely to help us gain greater insight into how existing social networks came into being. Recently, Abou-Zeid et al. (2013) reviewed the state of the art of social influence in transportation research, a field that has throughout history had a considerable impact on migration modelling. Multilevel models are the statistical counterpart to the study of networks. Apart from networks, other hierarchical levels, such as households or villages, can be included. Decision-making can occur at those levels, too. While agent-based models allow researchers to combine decision-making that occurs on different levels, this option has so far not been adequately exploited. This is a potentially fruitful avenue for future research.

Third, it is time to bridge the divide between microsimulation and agent-based modelling. The two methodologies are already being used jointly in several migration models (Massey and Zenteno 1999; Klabunde 2014; Mena et al. 2011). It is a task for future research to identify the criteria needed for determining whether a decision is best modelled as a rate or a probability (as in microsimulation) or as a rule (as in agent-based modelling).

Fourth, an innovative and systematic approach is required to validate ABMs. Table 2 in the Appendix reveals the great diversity in the degree of emphasis that is put on questions of validation, parameter estimation, and sensitivity analysis. Agent-based models allow researchers to test hypotheses “in the laboratory” to see which behaviours generate the observed outcomes. We argue in favour of using all of the available data to determine plausible parameter ranges and then to explore the parameter space extensively. A Gaussian emulator can be used to do this. The parameters most in line with the data can be estimated consistently using simulated minimum distance (Grazzini and Richiardi 2015). In most agent-based studies of migration, the sensitivity analysis, the estimation, and the validation are performed at rather rudimentary levels. We hope this will change in future work.

Fifth, ABMs of migration should position the migration decision in the human life course and use up-to-date life-history modelling techniques to describe how migration is intertwined with other life events. Over the past two decades, as more individual and longitudinal data have become available, demographic research has become increasingly oriented towards event history analysis. Simulation methods have been developed to describe the human life course and to predict life paths. Some authors use discrete event simulation techniques, whereas others rely on statistical methods for event history analysis. Future research should combine rigorous state-of-the-art event history analysis with simulation techniques.

Finally, there is a need for more applications of ABM in migration research. Many migration-related research questions can be tackled extremely well with agent-based modelling. Among these questions are the following: How do changes in social norms related to marriage and fertility affect the timing of migration? Do the size, direction, and timing of migration flows depend on whether the decision-making happens on the individual or on the household level? What proportion of migrant location decisions can be explained by social effects, and what proportion is attributable to other factors? What changes if the social influence is age-specific? Is the theory of planned behaviour a plausible decision theory for the migration decision? How will demographic change alter the composition of the migrant population? How will labour markets be affected?

Migration is a trending topic in demographic research, and its importance continues to increase (Bijak et al. 2014). Agent-based modelling of migration decision-making is just starting to develop. The initial attempts are promising and are paving the way for a new generation of models that can predict who will migrate, why they will migrate, and when they will migrate.

## Figures and Tables

**Table 3:**
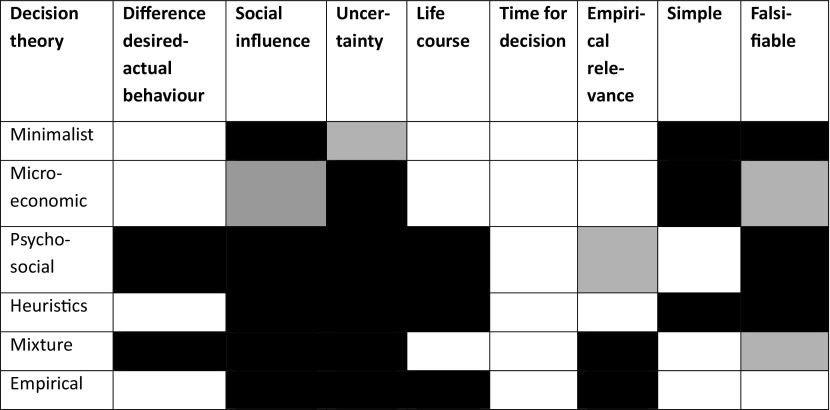
Evaluation of seven different decision theories according to eight evaluation criteria

White: not applicable, grey: somewhat applicable, black: applicable

## Appendix

See Tables 2 and 3.

**Table 2:** Other demographic aspects and use of data in agent-based models of migration

References	Demographic aspect	Estimation and/or calibration and/or validation and/or sensitivity analysis
Barbosa Filho et al. (2011)	None	Calibration (random values drawn from distributions estimated from data)
Berman et al. (2004)	Fertility, mortality, family composition, population ageing	Calibration through stakeholder interviews
Biondo et al. (2013)	None	Sensitivity analysis
Cai and Oppenheimer (2013)	None	Calibration of some parameters from different data sources. Estimation of other parameters within parameter ranges by minimising mean squared error
El Saadi et al. (2010)	Population growth	None
Espíndola et al. (2006)	Population growth	None
García-Díaz and Moreno-Monroy (2012)	None	Full factorial design: 3^6^ parameter combinations run. OLS as meta-model.
Hafizoglu and Sen (2012)	None	None
Hassani-Mahmooei and Parris (2012)	Net population growth, health	Parameter calibration from different sources. Out-of-sample validation. Estimation of three parameters
Heiland (2003)	None	Some parameters calibrated. Qualitative validation
Ichinose et al. (2013)	None	Sensitivity analysis varying one parameter at a time
Janssen (2010)	Fertility, mortality, population growth	Time-series meta-model on baseline model output. Sensitivity analysis: two values for some of the parameters; varied one at a time and some two-way interactions
Jiang et al. (2010)	None	None
Klabunde (2014)	Ageing	Calibration of parameters through survey data and different sources. Sensitivity analysis around fixed parameters. Estimation of three parameters through simulated minimum distance. Quantitative and qualitative output validation.
Kniveton et al. (2012)	Population growth	None
Kniveton et al. (2011)	Fertility, mortality, marriage, population ageing	Calibration from survey data; qualitative validation
Massey and Zenteno (1999)	Fertility, mortality	Calibration from survey and census data through discrete time event history methods
Mena et al. (2011)	Fertility, mortality, marriage	Calibration with survey and GIS data
Naivinit et al. (2010)	None	Interactive calibration and validation with stakeholders
Reichlová (2005)	None	None
Rehm (2012)	Mortality: life expectancy, fertility: number of children	Qualitative validation
Rogers et al. (2011)	Fertility, mortality	Qualitative validation; sensitivity analysis: varying one parameter at a time and two-way interactions
Schweitzer (1998)	None	None
Silveira et al. (2006)	None	Sensitivity analysis of some parameters: varied one-by-one, two-way and three-way interactions
Smajgl and Bohensky (2013)	None	Interactive calibration and validation with stakeholders
Smith (2014)	Fertility, mortality	Calibration with survey and climate data
Walsh et al. (2013)	Fertility, mortality, marriage	Calibration with survey data, agricultural data, and GIS data

## Footnotes

1 We assume the model described in both papers is identical as far as the decision-making is concerned; this is not clear because of the parsimonious model descriptions in both papers.
